# The Pathogenesis and Therapeutic Approaches of Diabetic Neuropathy in the Retina

**DOI:** 10.3390/ijms22169050

**Published:** 2021-08-22

**Authors:** Toshiyuki Oshitari

**Affiliations:** 1 Department of Ophthalmology and Visual Science, Chiba University Graduate School of Medicine, Inohana 1-8-1, Chuo-ku, Chiba 260-8670, Japan; Tarii@aol.com; Tel.: +81-43-226-2124; Fax: +81-43-224-4162; 2 Department of Ophthalmology, International University of Health and Welfare School of Medicine, 4-3 Kozunomori, Narita 286-8686, Japan

**Keywords:** neurovascular unit, neuronal cell death, glial abnormalities, axon degeneration, neuroprotection, axon regeneration, diabetic neuropathy, neurotrophic factors, intrinsic regenerative pathways

## Abstract

Diabetic retinopathy is a major retinal disease and a leading cause of blindness in the world. Diabetic retinopathy is a neurovascular disease that is associated with disturbances of the interdependent relationship of cells composed of the neurovascular units, i.e., neurons, glial cells, and vascular cells. An impairment of these neurovascular units causes both neuronal and vascular abnormalities in diabetic retinopathy. More specifically, neuronal abnormalities including neuronal cell death and axon degeneration are irreversible changes that are directly related to the vision reduction in diabetic patients. Thus, establishment of neuroprotective and regenerative therapies for diabetic neuropathy in the retina is an emergent task for preventing the blindness of patients with diabetic retinopathy. This review focuses on the pathogenesis of the neuronal abnormalities in diabetic retina including glial abnormalities, neuronal cell death, and axon degeneration. The possible molecular cell death pathways and intrinsic survival and regenerative pathways are also described. In addition, therapeutic approaches for diabetic neuropathy in the retina both in vitro and in vivo are presented. This review should be helpful for providing clues to overcome the barriers for establishing neuroprotection and regeneration of diabetic neuropathy in the retina.

## 1. Introduction

Diabetic retinopathy, a major complication of diabetic patients, is the leading cause of vision loss worldwide [1]. It has been predicted that approximately 600 million individuals aged 20–79 years will have diabetes in 2040 [1]. The results of an earlier study indicated that approximately 30% of diabetic patients have diabetic retinopathy and diabetic macular edema [2]. The META-EYE Study found that among 22,896 diabetic patients, 10.2% of the patients had vision-threatening diabetic retinopathy, i.e., proliferative diabetic retinopathy and/or diabetic macular edema [3]. In Japan, the prevalence of diabetic retinopathy in patients with type 2 diabetes was 23.5% in 2014 which is a significant decrease from the 31.1% in 2004. This was probably because of achieving treatment targets of reducing glycated hemoglobin A1c (HbA1c) from 7.46 ± 1.09% in 2004 to 7.00 ± 0.95% in 2014 [4]. However, younger patients with type 2 diabetes have difficulties in achieving treatment targets for HbA1c [4]. Thus, there are still concerns on the increase in the prevalence of diabetic retinopathy in younger patients in the future.

The clinical stages of diabetic retinopathy are diagnosed by the presence of vascular abnormalities such as retinal hemorrhages, microaneurysms, hard and soft exudates, non-perfused areas or neovascularization in the retina. Since 1998 [5], however, growing evidence has been demonstrating that neuronal abnormalities including neuronal cell death is related to the pathogenesis of the early stage of diabetic neuropathy in the retina in vitro [6,7,8,9,10,11,12,13,14,15,16], in vivo animal models [17,18,19,20,21,22,23,24,25,26,27,28,29,30,31,32,33,34,35], in human retinas [5,36,37,38], and in clinical studies from the findings of optical coherence tomography in patients with no or minimal diabetic retinopathy [39,40,41,42,43,44,45,46]. Although it is not determined yet, most studies demonstrated that neuronal abnormalities precede the clinical findings of vascular abnormalities. Certainly, subclinical microvascular changes may develop before neurodegeneration in some cases. However, growing evidence has demonstrated that diabetic retinopathy is not merely a vascular disease but is a more complicated neurodegenerative diseases. In fact, a recent definition of diabetic retinopathy by the American Diabetes Association is that diabetic retinopathy is a highly tissue-specific neurovascular complication of both type 1 and type 2 diabetes [47]. The impairment of the interdependence between multiple cell-types including neurons, glial cells and vascular cells in the retina is associated with the development and progression of diabetic retinopathy. This review focuses on neuroprotective and regenerative therapies for retinal ganglion cell neuropathy in diabetic retina. Although there are many studies demonstrating the neuroprotective therapies for diabetic neuropathy in the retina, few studies have reported on the regenerative therapies for diabetic neuropathy in the retina. Thus, most evidence of regenerative therapies introduced in this review are referenced in studies using other animal models including optic nerve injury models. Please note that this review does not focus on stem cell researches or neurogenesis because strategies of optic nerve regeneration consist of neuroprotection, axon elongation, and reconstitution of the optic nerve circuit. Hopefully, this review will be helpful for giving clues to establish regenerative therapies for diabetic neuropathy of the retina.

## 2. Pathogenesis of the Diabetic Retinopathy: The Impairment of the Neurovascular Unit

**Figure 1:**
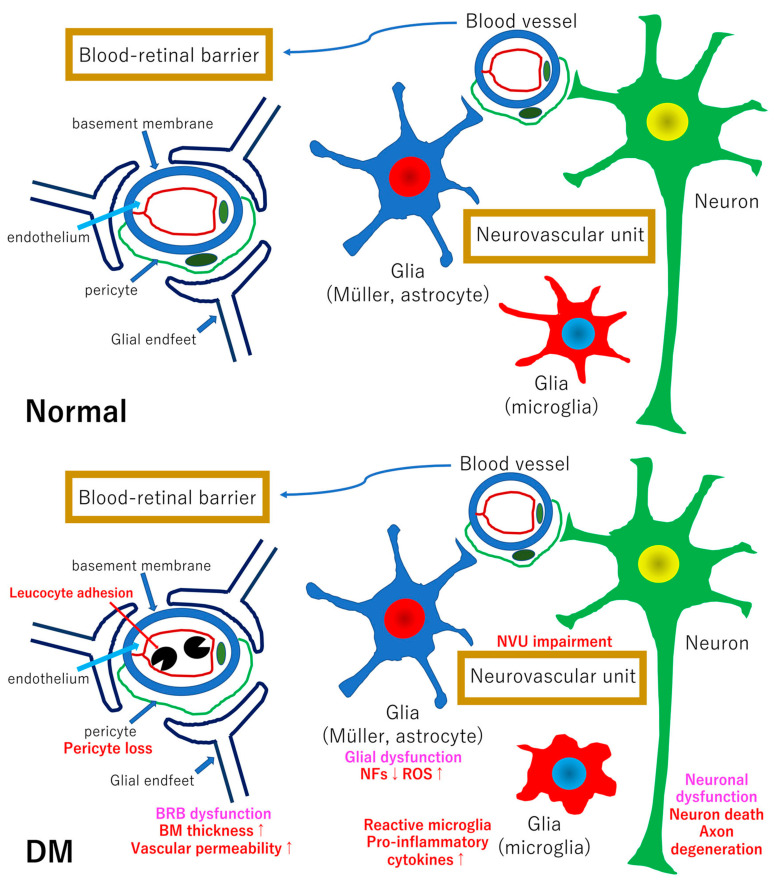
Hypothetical schemes of neurovascular units in normal and diabetic retinas. The upper panel shows the neurovascular unit of the normal retina. Müller cells and astrocytes are involved in the construction of the blood–retinal barrier (BRB) because the endfeets of glial Müller cells and astrocytes are one of the components to maintain the BRB function. However, it was recently reported that microglia to be members of the neurovascular unit. For example, healthy neurons release fractalkine (CX3C ligand 1), which binds to its CX3C chemokine receptor 1 on microglia and regulate microglia repopulation followed by maintaining the retinal homeostasis [53]. Pericytes release platelet-derived growth factor (PDGF) which maintain the BRB, and the loss of PDGF signaling in pericytes may cause neuronal cell death [54]. The lower panel shows the neurovascular unit of diabetic retina. Blood-circulating leukocytes adhere to the surface of endothelial cells via intracellular adhesion molecule-1 and vascular cell adhesion molecule which may cause to the occlusion of capillaries, leukostasis. The loss of pericytes is an early signs of microvascular change which may result in microaneurysm formation. Tight junction protein downregulation [55] and basement membrane thickening are associated with vascular permeability [56]. These changes in the vessels are monitored by glial cells followed by Müller cells or astrocyte dysfunction and microglia activation. Activated microglia releases a variety of inflammatory cytokines such as tumor necrosis factor-α (TNF-α), interleukin-1β (IL-1β), interleukin-6 (IL-6), -8 (IL-8), and monocyte chemoattractant protein-1 (MCP-1). Glial dysfunction may lead to a reduction in the release of neurotrophic factors such as nerve growth factor (NGF), brain-derived neurotrophic factor (BDNF), or PDGF. These glial changes contribute to neuronal cell death and axon degeneration. Glial changes may occur first and thus, neuronal abnormalities may occur faster than the vascular abnormalities in the clinical findings. DM: diabetes mellitus, BM: basement membrane, NVU: neurovascular unit, NFs: neurotrophic factors, ROS: reactive oxygen species.

In the retina, all cells communicate each other and maintain healthy retinal environment and function. The anatomical and functional interdependence of neurons, glial cells and vascular cells has been proposed as neurovascular units, and the impairment of neurovascular units are thought to be primary pathological changes in early diabetic retinopathy [48]. The hypothetic scheme of neurovascular units in normal and diabetic retinas are shown in Figure 1. Neurovascular units are composed of neurons (ganglion cells, amacrine cells, horizontal cells, and bipolar cells), glial cells (Müller cells, microglia, and astrocytes) and vessels (endothelial cells, pericytes, and basement membrane) [48]. The inner blood–retinal barriers are composed of tight junction of endothelial cells, basement membrane, and glial endfeets (Figure 1). A representative example of the interdependence between cells involving the neurovascular units in normal conditions is functional hyperemia [49]. Briefly, activated neurons release glutamate, K^+^, and adenosine triphosphate (ATP) from the synapses, followed by stimulation of glial cells (astrocytes). In the stimulated astrocytes, inositol triphosphate is produced and intracellular Ca^2+^ levels are increased. High concentration of Ca^2+^ results in the activation of phospholipase A_2_ (PLA_2_) which then releases arachidonic acid (AA) from membrane phospholipids. AA is metabolized to epoxyeicosatrienoic acids and prostaglandin E_2_ which result in dilation of the vessels [50]. In cases of AA being converted to 20-hydroxy-eicostatrenoic acid, vessels are constricted [50].

Disturbances of the interdependence among cells involved in the neurovascular units are associated with the pathogenesis of the early stage of diabetic retinopathy. Chronic hyperglycemia can be a trigger for the impairment of neurovascular units via intracellular excess glucose flux followed by overproduction of reactive oxygen species (ROS) and advanced glycation-end products (AGEs) [51]. AGEs can be sources for ROS production and ROS is associated with mitochondrial dysfunction, resulting in the disturbance of the blood barrier function and the impairment of neuronal tissue homeostasis [52].

Pericytes are essential for the BRB formation and maintenance of homeostasis of vascular system in the retina and the brain [57]. PDGF subunit B signaling to pericytes is essential for maintaining a homeostasis of the BRB [58]. The results of this study indicate that pericytes regulate angiopoietin-2 and vascular endothelial growth factor (VEGF) receptor-2 in endothelial cells via transcription factor Forkhead box protein O1 [56]. In experimental models of diabetic retinopathy, the loss of pericytes occurs before endothelial cell loss [59]. Thus, pericyte loss in early diabetic retinopathy causes an increase of VEGF signaling followed by an increase of vascular permeability and endothelial cell loss.

In the early stage of diabetic retinopathy, Müller cell abnormalities have been observed including increasing expression of glial fibrillar acidic protein (GFAP) [60], swelling of Müller cells [61], and amplifying pro-inflammatory reactions of activated microglia through P2X_7_ purinergic receptors [62]. On the other hand, the number of astrocytes and GFAP expression in astrocytes are decreased in the early stage of diabetic retinopathy [63]. Although GFAP is a marker for reactive gliosis, studies have not demonstrated the relationship between glial dysfunction and GFAP expression. However, these early glial changes may be related to the impairment of the neurovascular units in early diabetic retinopathy. Antonetti et al. describe in a recent review that aquaporins and Kir4.1 channels in glial cells are increased followed by glial swelling and production of VEGF-A, Delta-like protein 4, and angiopoietin-related protein 4 in diabetic retinas, [64]. These cytokines are known to promote vascular permeability and angiogenesis. In fact, Wang et al. demonstrated that Müller cell derived VEGF is essential for retinal inflammation and vascular leakage in diabetic retinopathy by using VEGF knockout in Müller cells [65]. On the other hand, Matteucci et al. reported that Müller cell activation was not the cause to neurodegeneration but contributed to neuroprotection though extracellular signal-regulated kinase 1/2 (ERK1/2) activation both in vitro and in vivo high glucose exposure experiments [66].

Microglia, one of the components of the neurovascular units, are also involved in the pathogenesis of diabetic retinopathy. Microglia have two activated phenotypes, the pro-inflammatory (M1) state and the anti-inflammatory (M2) state [67]. For example, the pro-inflammatory microglia secret proinflammatory cytokines such as IL-1β, IL-6, IL-8, and TNF-α. The anti-inflammatory forms secret cytokines such as IL-4, IL-10, IL-13, and transforming growth factor-β. The results of many studies have indicated that the anti-inflammatory state shifts to the pro-inflammatory state in diabetes [68,69,70]. These shifts are observed before the neuronal cell death in the diabetic retina [68]. Chronic hyperglycemia causes an increase in the AGEs which stimulate the expression of TNF-α in microglia through ERK and nuclear factor kappa B (NF-κB) [71]. The hyperglycemia can also induce VEGF expression in microglia through the ERK1/2-NF-κB signaling pathway [72]. Thus, reactive microglia are stimulated by chronic hyperglycemia and are associated with the progression of the diabetic retinopathy.

Neuronal cell death and axon degeneration are directly linked with the vision reduction in patients with diabetic retinopathy. Most basic and clinical studies have shown that neuronal dysfunction and neuronal cell death precedes the clinical findings of vascular abnormalities and thus, the onset of neuronal dysfunction and neuronal cell death is not the results of vascular abnormalities [5,7,39,40,41,42,43,45,46,73,74,75,76,77]. Retinal ganglion cells and amacrine cells appear to be more sensitive to diabetic stress than photoreceptors in the early stages of diabetic retinopathy probably because retinal vessels exist in the inner retinal layers and the impairment of the neurovascular units is involved in the neuronal cell death in the inner retina. Neuronal cell death is an irreversible change and it is directly related to vision reduction in patients with diabetic retinopathy. In addition, not only neuronal cell death but also axon degeneration are associated with the pathogenesis of diabetic retinopathy [39,40,41,42,43,44,45,46]. For example, in early diabetic retinopathy, the thicknesses of the nerve fiber layer, ganglion cell layer, and the inner plexiform layer are reduced by 0.54 μm per year which is almost equal to the reduction found in patients with severe glaucoma [43]. In addition, the results of several studies have indicated that the axonal transport is disturbed in diabetic animal models [78,79,80]. The retinal ganglion cells (RGCs) and optic nerve belong to the central nervous system. Once their axons are degenerated, they cannot regenerate under physiological conditions because their intrinsic survival and regenerative abilities are decreased and the glial environment of the central nervous system disturbs axonal regeneration [81].

## 3. Multimodal Approaches for Axonal Protection and Regeneration

**Figure 2:**
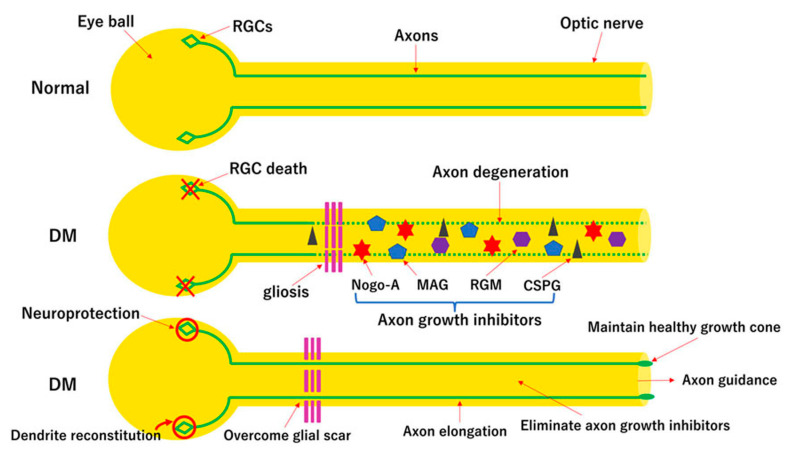
Scheme of multiple approaches for optic nerve regeneration in diabetic retina. RGCs are vulnerable under diabetic stress (the middle diagram). Therefore, in the early stage, RGCs must be protected. At the same time, however, axons are also degenerated. Thus, degenerated axons must also be regenerated. However, the glial environment of the central nervous system suppresses axonal regeneration because of astrogliosis and myelin debris such as Nogo-A or myelin-associated glycoprotein (MAG). For the success of optic nerve regeneration, the RGC death must be blocked and regenerating axons must overcome the glial scar barriers and elongate their axons in the middle of axon growth inhibitors. To overcome these barriers, the growth cone must be maintained in a healthy condition. Finally, regenerating axons must connect to the correct target cells in the brain by regulating axon guidance cues. DM, diabetes mellitus; RGM, repulsive guidance molecule; CSPG, chondroitin sulfate proteoglycan.

RGCs and their axons are more vulnerable than other neurons in diabetic retina [39,40,41,42,43,44,45,46] and thus, optic nerve regeneration is required for functional recovery in eyes with diabetic neuropathy of the retina. For that purpose, multiple approaches including increasing the intrinsic survival and regenerative abilities and overcoming the inhibitory glial environment are required. A scheme of multiple strategies for optic nerve regeneration is shown in the Figure 2.

### 3.1. Mechanisms of Neuronal Cell Death in RGC Neuropathy in Diabetic Retina

**Figure 3:**
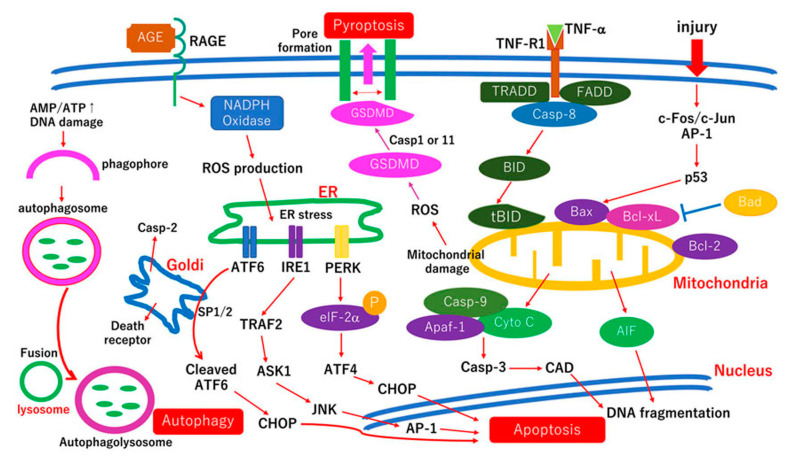
Hypothetic cell death pathways of RGCs under diabetic stress. Diabetic stress includes hyperglycemia, AGEs signaling, oxidative stress, excitotoxicity, and polyol pathway. These biochemical injuries are similar to physical injuries. In fact, some cell death mechanisms of biochemical injuries are, in part, common with those of physical injuries. Thus, the scheme partly includes evidence derived from optic nerve injury models and retinal culture studies. In cultured retinas, c-Fos is essential for neuronal apoptosis after injury [82]. C-Fos transfers the cell death signals to mitochondria by p53 upregulation resulting in Bax upregulation and Bcl-xL downregulation [82]. C-Jun is a partner of c-Fos and makes heterodimer activator protein-1 (AP-1). Retinas exposed to high glucose activate c-Jun and JNK in neurons in the ganglion cell layer [13]. Thus, the c-Fos/c-Jun (AP-1)/JNK signaling axis is related to neuronal cell death under diabetic stress [13]. Caspase -8, -9, and -3 are associated with neuronal cell death [83]. In fact, overexpression of Bcl-xL rescues damaged retinal ganglion cells [84]. In AGEs exposed retina, caspase-9, and apoptosis-inducing factor (AIF) expression are correlated with increasing neuronal cell death [11]. Thus, both caspase-dependent and caspase-independent pathways are associated with the AGE-induced neuronal cell death [11]. The AGE-RAGE signaling results in increasing the production of ROS which leads to ER stress-induced apoptosis. In fact, taurine-conjugated ursodeoxycholic acid (TUDCA) and anti-ER stress reagents reduce neuronal cell death in AGE-exposed retinas [16]. ROS production is also associated with pyroptosis via cleavage of GSDMD by caspase-1 or -11. Autophagy signaling is usually regulated by the mammalian target of rapamycin (mTOR) pathway under physiological conditions. Under diabetic stress, the intrinsic survival pathway should be decreased resulting in activation of autophagy signaling. Over activation of autophagy signaling may cause to autophagic cell death. TNF-α, tumor necrosis factor-α; TRADD, TNF receptor 1-associated death domain protein; FADD, Fas-associated death domain; TRAF2, TNF receptor-associated factor 2; Casp8, caspase-8; tBID, truncated Bid; Bcl-2, B-cell lymphoma 2; Bcl-xL, B-cell lymphoma-extra large; Casp-9, caspase-9; Apaf-1, Apoptosis protease-activating factor 1; cyto c, cytochrome c; ROS, reactive oxygen species; GSDMD, Gasdermin D; CAD, Caspase-activated DNase; AP-1, activator protein-1; ER, endoplasmic reticulum; PERK, protein kinase-like ER eukaryotic initiation factor-2alpha kinase; IRE1, inositol-requiring ER-to-nucleus signaling protein 1; ATF6, activating transcription factor-6; CHOP, CCAAT/enhancer-binding protein homologous protein; JNK, c-Jun *N*-terminal kinase; elF-2α, Eukaryotic Initiation Factor-2α; AGEs, advanced glycation end-products; RAGE, receptor for AGEs; NADP, nicotinamide adenine dinucleotide phosphate (NADP+ oxidized form; NADPH reduced form); AMP, adenosine monophosphate, ATP, adenosine triphosphate.

As described above, the RGCs are more vulnerable than other neurons in diabetic retina. Therefore, damaged RGCs must be protected under diabetic stress. To establish neuroprotective therapies, neuronal cell death mechanisms in damaged RGCs in the diabetic retina must be determined. The hypothetic cell death pathways of damaged RGCs are shown in the Figure 3.

The results of several human retinal studies have indicated that in human retinal sections obtained from diabetic patients, apoptotic cell death markers have been identified in mainly neurons in the ganglion cell layer [36,37,85,86] including c-Fos/c-Jun (AP-1) [37], JNK [37], Bax [36,86], caspase-9 [36], caspase-3 [36,86], and Bad [85]. AIF and cytochrome c are detected in the photoreceptors [85]. More specifically, c-Fos/c-Jun, JNK, Bax, active form of caspase-9, and active form of caspase-3 signals coexisted with the expression of Fluoro-Jade B, degenerative neuronal markers in the ganglion cell layer in diabetic retina in our human retinal studies, [36,37]. These results indicated that JNK/AP-1 signaling and the mitochondria- and caspase-dependent cell death pathways are activated in degenerating neurons in the ganglion cell layer of diabetic retinas [36,37].

We have performed studies to determine the mechanisms of neuronal cell death and to establish neuroprotective and regenerative therapies by using three-dimensional collagen gel culture systems [7,9,10,11,13,14,15,16,82,83,84,87]. A previous study indicated that the retina in *c-fos* deficient mice had some apoptotic cell death that was observed after 14 days in culture. On the other hand, in the retina of littermates, 90% of the neuronal cells in the ganglion cell layer died by apoptosis [82]. Furthermore, in *c-fos* deficient retinas, p53 and Bax expressions were decreased but Bcl-xL expression was increased [82]. These results demonstrated that c-Fos is essential for retinal neuronal cell death in the ganglion cell layer, and that the c-Fos transferred the cell death signals to the mitochondria [82]. The results of another study indicated that caspase -3, -8, and -9 inhibitors rescued damaged retinal neurons in this culture system [83]. The maximum rescue ratio was 60% and thus, 40% of retinal ganglion cells died in a caspase-independent manner [83]. In fact, AIF expression was significantly increased in AGE-exposed retinas compared to normal retinas [11]. Thus, both caspase-dependent and -independent pathways are associated with neuronal cell death under diabetic stress [11]. In retinas cultured in high glucose medium, phosphorylated c-Jun, and phosphorylated JNK expression in neurons in the ganglion cell layer were significantly increased compared to retina cultured in normal glucose medium [13]. Thus, c-Jun/JNK signaling is associated with neuronal cell death under diabetic stress [13]. These apoptosis related factors observed in this culture system under diabetic stress are also found in degenerating neurons in human diabetic retinas [36,37,86] (Figure 3). Taken together, the cell death mechanisms involved in neuronal cell death of cultured retina under diabetic stress are partly common to those in the neuronal cell death in the human retinas of diabetic patients.

We have examined the neuroprotective and regenerative effects of several neurotrophic factors in this culture system under diabetic stress. Briefly, neurotrophin-4 (NT-4), brain-derived neurotrophic factor (BDNF), glial cell line-derived neurotrophic factor, hepatocyte growth factor, TUDCA, and citicoline showed neuroprotective and regenerative effects in retinas cultured under diabetic stress [9,10,11,13,14,15,16]. Among these neurotrophic factors, NT-4 shows the most neuroprotective and regenerative effects. In high glucose exposed retina, TUDCA, an anti-ER stress reagent, had comparable neuroprotective effects to NT-4, but the regenerative effect of NT-4 had more than that of TUDCA [13]. These findings suggested that the phenomenon of regeneration is different from that of survival, and that not all neuroprotective therapies are sufficient for supporting optic nerve regeneration. Thus, therapeutic strategies other than neuroprotection are required for optic nerve regeneration.

Oxidative stress including overproduction of ROS results in ER stress related cell death or pyroptosis (Figure 3) and noncoding RNAs are involved in alteration of ROS homeostasis induced by oxidative stress [52]. Oxidative stress induces the alteration of several transcription factors such as AP-1, p53, or NF-κB which are involved in immune response, growth factor signaling, and neurodegeneration [52]. These possible involvements of epigenetic mechanisms induced by oxidative stress are also important, but go beyond the scope of this review.

### 3.2. Intrinsic Survival and Regenerative Pathways for Optic Nerve Regeneration

**Figure 4:**
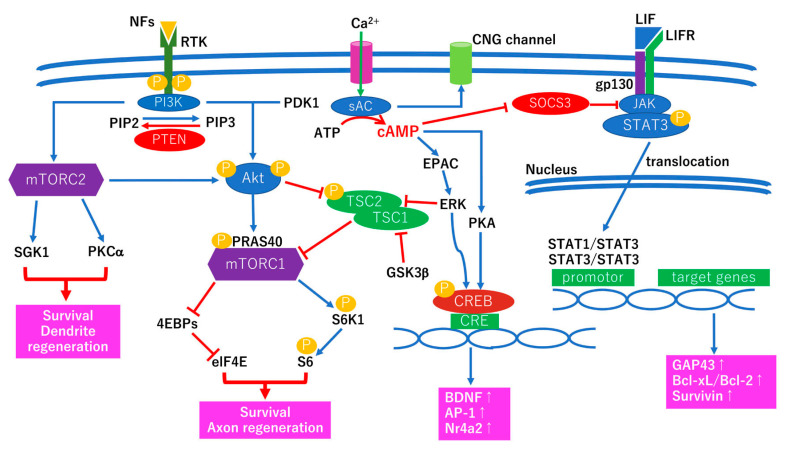
Simple scheme of the intrinsic survival and regenerative pathways. The figures include only two major pathways; the mTOR pathways and the JAK/STAT pathways. In addition, not all cAMP signaling pathways are included because of the limited space. First, the neurotrophic factors (NFs) such as BDNF bind with their receptors and receptor tyrosine kinase (RTK) is activated. Then, PI3K is phosphorylated resulting in an increase of PIP3 accumulation. Phosphatase and tensin homolog (PTEN) are inhibitors of the mTOR pathway [89]. PTEN dephosphorylates PIP3, resulting in the accumulation of PIP2. The accumulated PIP3 recruits PDK1 and the corresponding residue (T308) of Akt kinase is phosphorylated. At the same time, the other residue (Thr308) of Akt kinase is phosphorylated by mTOR complex 2 (mTORC2). A full activated Akt by double phosphorylation by PDK1 and mTORC2 inhibits TSC2 which results in converting Ras homolog enriched in brain (Rheb)-GTP to Rheb-GDP. The TSC1/2 complex inhibits the mTOR complex 1 (mTORC1). Thus, the inhibition of mTORC1 via TSC1/2 complex is eliminated by the Akt signaling pathway. Eukaryotic translation initiation factor 4E-binding proteins (4EBPs) bind tightly to eukaryotic initiation factor-4E (eIF4E). Activated mTORC1 dislocates two proteins, resulting in relieving their inhibition of the initiation of protein synthesis. Both 4EBPs inhibition and S6K1 activation facilitate adult optic nerve regeneration [90]. On the other hand, activated mTORC2 is thought to promote the dendrite extension and the reestablishment of the arbor area [91]. Another major intrinsic survival and regenerative pathway is the JAK/STAT pathway. The JAK/STAT pathway is activated by several stress-related cytokines such as IL-6 family cytokines including IL-6, ciliary neurotrophic factor (CNTF), and leukemia inhibitory factor (LIF). The figure shows that LIF and their receptor subunits, glycoprotein 130 (gp130) and LIF receptor. Gp130, are common receptor subunits of the IL-6 family cytokines and after binding with these cytokines, the JAK/STAT pathway is activated. The intrinsic inhibitor of the JAK/STAT pathway is a suppressor of cytokine signaling 3 (SOCS3) [92]. After activation of JAK, STAT family members make homodimers or heterodimers which regulate specific genes involved in the survival and regeneration of the optic nerve axons. The cAMP signaling is activated by electrical activity and enhances the regenerative effect of several neurotrophic factors [93]. The downstream of cAMP signaling includes protein kinase A (PKA) and exchange protein activated by cAMP (EPAC). The precise mechanisms of cAMP signaling is still unclear but electrical activity may stimulate cAMP synthesis and facilitate axonal regeneration after injury [94]. NFs, neurotrophic factors; RTK, receptor tyrosine kinase; PI3K, phosphatidylinositol-3 kinase; PIP2, phosphatidylinositol (4,5)-bisphosphate; PIP3, phosphatidylinositol (3,4,5)-triphosphate; PTEN, phosphatase and tensin homolog; PDK1, phosphoinositide-dependent protein kinase 1; mTORC1, mammalian target of rapamycin complex 1; PRAS40, protein-rich Akt substrate of 40kDa; S6K1, ribosomal protein S6 kinase 1; TSC1/2, tuberous sclerosis complex1/2; GSK3β, glycogen synthase kinase-3 beta; 4EBPs, eukaryotic translation initiation factor 4E-binding proteins; elF4E, eukaryotic initiation factor-4E; mTORC2, mammalian target of rapamycin complex 2; SGK1, serum and glucocorticoid-regulated kinase 1; PKCα, protein kinase C-alpha; sAC, soluble adenylyl cyclase; cAMP, cyclic adenosine monophosphate; EPAC, exchange protein activated by cAMP; PKA, protein kinase A; ERK, extracellular signal-regulated kinase; CRE, cAMP response element; CREB, CAMP response element binding protein, BDNF; brain-derived neurotrophic factor, AP-1; activator protein-1, CNG channel; cyclic nucleotide gated channel, Nr4a2; nuclear receptor subfamily 4 group a member 2, LIF; leukemia inhibitory factor, SOCS3; suppressor of cytokine signaling 3; JAK, Janus kinase; STAT, signal transducers and activators of transcription; GAP43, growth associated protein 43; gp130, glycoprotein 130; LIFR, LIF receptor.

To achieve a successful optic nerve regeneration, the intrinsic survival and regenerative pathways needs to be understood. The intrinsic survival and regenerative abilities are known to be decreased in neurons in the central nervous system after damage. The first step of optic nerve regeneration is to increase the abilities of the intrinsic survival and regenerative pathways [88]. The hypothetic pathways for intrinsic survival and regenerative pathways are show in the Figure 4.

The two major intrinsic survival and regenerative pathways are the PI3K/Akt/mTOR pathway [89] and the JAK/STAT pathway [92] (Figure 4). The mTOR pathway is a well-known intrinsic survival and regenerative pathway, and PTEN is a negative regulator of the mTOR pathway [89]. Deletion of PTEN [89] or PTEN knockdown by a tyrosine-mutated adeno-associated virus serotype 2 vector [95] promotes the survival and robust axon regeneration after optic nerve injury. The major factor in the upstream of the mTOR pathway is Akt kinase. Akt hast two phosphorylated sites, S473 and Thr308. PDK1 phosphorylates Akt on the T308 site [96] and mTORC2 phosphorylates Akt on the S473 site [97]. Fully activated Akt activates mTOR by at least two different pathways. One is the TSC1/2–Rheb pathway [98] and the other is the PRAS40 pathway [99]. However, the role of PRAS40 phosphorylation in the mTOR pathway is still being debated. Thus, the TSC1/2-Rheb seems to be a major upstream circuit of the mTOR pathway. The results of a recent study indicated that one of the components of mTORC1 and mTORC2, regulatory associated protein of mTOR (RAPTOR) and rapamycin-insensitive companion of mTOR (RIPTOR), are localized in the inner retina of human eyes [100]. More specifically, RAPTOR is expressed in the retinal ganglion cells and RIPTOR is expressed in glial cells [100]. Furthermore, the S6K1 expression in RGCs indicated that the mTORC1 signaling is preserved in human retinas [100]. Thus, the intrinsic survival and regenerative pathways may be activated under physiological conditions to regulate the homeostasis of retinal function.

Fully activated Akt is involved in many cellular events including survival and regeneration. Downstream effectors of activated Akt include mTORC1, endothelial nitric oxide synthase (eNOS), Bad, caspase-9, glycogen synthase kinase-3β (GSK3β), and murine double minute 2 (MDM2) [81]. The PI3K/Akt/eNOS pathway is related to a neuroprotection of gardenamide A against H_2_O_2_ exposure-induced retinal ganglion cell death [101]. Phosphorylated Bad [102] and caspase-9 [103], activated Akt, result in an inhibition of the mitochondria-caspase dependent cell death pathway. This pathway is associated with neuronal cell death in human diabetic retinas [36]. The results of a recent study indicated that one of the microtube-binding proteins, collapsing response mediator protein 2 (CRPM2), is inactivated by GSK3 in retinal ganglion cell axons and that inhibition of GSK3 facilitates axon regeneration via constitutive activation of CRPM2 [104]. Joshi et al. reported that Nutrin-3, an antagonist of MDM2-p53 interaction, promoted the corticospinal axon regeneration [105]. Taken together, activated Akt plays a critical role in neuroprotection and regeneration. However, the results of a recent study suggested the existence of a PTEN-dependent pathway and a Akt-independent pathway in PTEN knockout mice [106]. Further studies are required to determine the intrinsic survival and regenerative pathways.

Another intrinsic survival and regenerative pathway is the JAK/STAT pathway, and a key suppressor is SOCS3 [92] (Figure 4). The JAK/STAT pathway is activated by several stress-related cytokines including IL-6 [107], CNTF [108], and LIF [109]. A common receptor component of the IL-6 family is gp130 and after binding cytokines, the JAK/STAT pathway is activated [107]. The major effectors of the JAK/STAT pathway, STAT1 and 3, make a heterodimer or a homodimer and are translocated into the nucleus followed by regulating gene expression of GAP43 [110], Bcl-xL/Bcl-2 [111], and survivin [112] (Figure 4). Deletion of both PTEN and SOCS3 promotes more axonal regeneration after optic nerve injury than a single deletion of PTEN or SOCS3 [113]. Thus, the mTOR pathway suppressed by PTEN is independent of the JAK/STAT pathway suppressed by SOCS3 [113].

Other intrinsic regenerative genes and pathways include Wnt signaling [114], Lin28 [115], Sry-related high-mobility-box 11 [116], Krüppel-like factor 4/9 [117], histone deacetylase [118], c-myc [119], and cAMP [93,120]. However, a single manipulation of these pathways or these genes is not enough for promoting long-lasting optic nerve regeneration in vivo. Thus, multiple approaches are required for long-lasting optic nerve regeneration.

### 3.3. Multimodal Strategies for Optic Nerve Regeneration

To overcome the inhibitory environment for optic nerve regeneration in the central nervous system, multimodal approaches have been performed. De Lima et al. used a combination therapies of deletion of PTEN with intravitreal injection of zymosan and a cAMP analog, and they successfully achieved long distant optic nerve regeneration beyond the optic chiasma after an optic nerve crush [121]. Furthermore, a partial functional recovery was obtained by evaluating the optomotor responses, depth perception, and circadian photoentrainment [121]. However, Luo et al. demonstrated by using tissue clearance and light sheet fluorescence microscopy that in PTEN deleted/Zymosan/cAMP mice, many regenerating axons go in the wrong direction after the optic chiasma [122]. Li et al. used a double deletion of PTEN/SOCS3 combined with CNTF injection therapies and successfully achieved long distant optic nerve regeneration beyond the optic chiasma and a partial functional recovery was obtained [123]. However, both the innervation pattern and evoked potentials were not completely restored in the regenerating axons [123]. These studies indicated the need for adequate axonal guidance for regenerating axons to achieve full functional recovery after injury.

However, Lim et al. suggest that a combination of Rheb1 overexpression with visual stimulation successfully promoted the longest axonal regeneration beyond the optic chiasma [94]. Surprisingly, after the optic nerve chiasma, the regenerating axons ran in the correct direction and reached the visual cortex [94]. One reason for the success is that visual stimulation may increase intracellular cAMP level [93] (Figure 4). Another possible reason is that electrical stimulation may be similar to neuronal physiological activities resulting in stabilizing the growth cone formation in the healthy condition. Thus, mimicking the physiologically healthy activities of regenerating neurons may be one of clues to overcome the inhibitory environment in the central nervous system for axonal regeneration.

The results of a recent study indicated that a knockout of myosin IIA and IIB facilitated robust axonal regeneration after optic nerve crush because pathological growth cones which make the retraction bulb formation was changed into healthy growth cones [124]. As a result, U-turned regenerating axons were reduced in the optic nerve. Because myosin IIA/IIB knockout is independent of the mTOR pathway, this strategy can be combined with the classical approaches for axon regeneration [124]. The blebbistain derivative pharmacologically inhibited myosin II [125] and thus, myosin II inhibition can be one of the options for the translational therapies for optic nerve regeneration.

For the translation of the basic studies into the clinical practice, the results of gene mutant mice are still far from the reality. One of the translational research is that instead of PTEN knockout, human peptide-based targeting of *C*-terminal PTEN PSD95/Dlg1/ZO-1 homology interactions are used for functional recovery after central nervous system injury [126]. Furthermore, some drug systems including thermosensitive hydrogel to deliver CNTF and FK506 [127], sulfonated reverse thermal gel to deliver CNTF [128], liposomes carrying multiple signal pathway modulators in retinal ganglion cells [129] promotes survival, regeneration, and functional recovery after damage. The designer cytokine, hyper-IL-6 (hIL-6), facilitates more neurite regeneration than CNTF or IL-6 and hIL-6 activates both the JAK-STAT and the mTOR pathways [130]. The adeno-associated virus delivered hIL-6 promotes robust axon regeneration beyond the optic chiasma [130]. Topical administration of a highly purified recombinant human NGF facilitates the survival and regeneration of RGCs [131], and topical application of NGF has been already used for patients with retinitis pigmentosa in a clinical pilot study [132]. Although the reconstitution of the optic nerve circuit is the last barrier, the regeneration of the optic nerve is not far from realization in the clinical practice.

## 4. Neuroprotective Therapies for Diabetic Retinopathy

Diabetic retinopathy is a chronic retinal disease and neuroprotective and regenerative therapies are tolerated and they can have chronic use. Therefore, topical instillation of neuroprotectants has been used in the first clinical trial in the world [133]. The EUROCONDOR study used topical application of somatostatin or brimonidine for patients with diabetic retinopathy [133]. A total of 450 patients with type 2 diabetes were enrolled at 11 European centers. Patients with no apparent retinopathy or mild non-proliferative diabetic retinopathy were included. The primary end point was the changes in the implicit time assessed by multifocal electroretinography. Unfortunately, the randomized multicenter-based clinical trial failed to reach the primary end point [133]. Possible reasons for the failure are the short study period and the low sensitivity of multifocal electroretinography. A longer period of study and the use of microperimetry-based evaluation might have obtained different conclusions in the EUROCONDOR study. However, in the patients with preexisting retinal neurodysfunction, the implicit time worsened in the placebo group and remained unchanged in the treatment group [133]. Thus, further clinical trials similar to the EUROCONDOR study must be performed repeatedly.

However, before planning further clinical trials, clinicians must reconsider the reasons for the failure of this study. Diabetic retinopathy is a chronic retinal disorder, and the cell death mechanisms should be more complicated than the acute injury models. Thus, a single neuroprotectant treatment may not be sufficient for protecting damaged neurons in diabetic retinopathy. In addition, axons of the retinal ganglion cells are known to degenerate in the early stages of diabetes. Thus, regenerative medicine should be included in the treatment options.

As described above, multimodal approaches are required for functional recovery after axon degeneration. For this reason, we selected three neuroprotectants; citicoline, TUDCA, and NT-4 and examined the effects of a combination of the three neuroprotectants on survival and regeneration in AGEs-exposed retinas [16]. A combination of these three agents had the most neuroprotective and regenerative effects compared to that of the single agent treated groups [16]. We used the optic nerve crush model and topical instillation of these three agents was performed for evaluating the neuroprotective and regenerative effect in vivo [134]. Citicoline is an intermediate product in the biosynthesis of phosphatidylcholine, and it reduces the degradation of cardiolipin by indirectly inhibiting PLA_2_ [135]. Thus, citicoline may be a mitochondria stabilizer. TUDCA is an anti-ER stress agent and protects neuronal cells from ER-stress related cell death [136]. NT-4 is the most neuroprotective and regenerative agent in vitro [9,10,11,13,14,15,16], and it activates the mTOR pathways contributing to myelination [137], attenuating neuroinflammation [138], and mediating neurogenesis [139]. The results of our study indicated that the combined topical application of these three agents had the most neuroprotective and regenerative effect after optic nerve crush in vivo [134]. Thus, such a combined topical application of neurotrophic factors may be effective for diabetic retinopathy. Further studies are required to examining the combined topical application of neurotrophic factors in diabetic animal models.

## 5. Conclusions

Diabetic retinopathy is a highly tissue-specific neurovascular complication of both type 1 and type 2 diabetes, and the impairment of neurovascular unit is associated with the early stages of diabetic retinopathy. Neuronal cell death and axon degeneration are observed from the early phase of diabetic retinopathy and these irreversible changes are directly related to vision reduction. Thus, not only neuroprotection but also axonal regeneration are required to prevent the progression of the neuronal abnormalities. However, single agent treatment may not be sufficient because of the complexity of the pathological changes in diabetic neuropathy of the retina. Multiple strategies for axonal protection and regeneration may be required even in the early stage of diabetic retinopathy. Further multimodal approaches for axonal regeneration should be considered before planning further clinical trials. Hopefully, this review gives a clue to establish a new multimodal therapy for diabetic neuropathy of the retina.
